# Target and off-target effects of vibegron on smooth muscle contraction of human detrusor and prostate tissues

**DOI:** 10.1007/s00210-025-04762-z

**Published:** 2025-11-03

**Authors:** Sheng Hu, Yajie Xu, Michael Brandstetter, Alexander Tamalunas, Oluwafemi E. Kale, Patrick Keller, Leo Federico Stadelmeier, Philipp Weinhold, Christian G. Stief, Martin Hennenberg

**Affiliations:** 1 Department of Urology, LMU University Hospital, LMU Munich, Munich, Germany; 2 Urologische Klinik und Poliklinik, Marchioninistr. 15, 81377 Munich, Germany

**Keywords:** Lower urinary tract symptoms (LUTS), Overactive bladder (OAB), Benign prostatic hyperplasia (BPH), Smooth muscle contraction, β_3_-adrenoceptor, Vibegron

## Abstract

**Purpose:**

The β_3_-agonist vibegron is available for treatment of storage symptoms, but in vitro data in human tissues are not yet available. Based on findings with mirabegron, mechanisms other than inhibition of voiding contractions in the detrusor may account for symptom improvements, and off-target effects in the prostate and bladder appear possible. Here, we examined vibegron effects in human detrusor and prostate tissues.

**Methods:**

Detrusor and prostate tissues were obtained from radical cystectomy, radical prostatectomy and laser enucleation. Concentration response curves for agonists and frequence response curves for electric field stimulation (EFS) were examined in organ baths with 0.01, 0.1, 1 and 10 µM vibegron or vehicle control.

**Results:**

In detrusor tissues, 10 µM vibegron reduced EFS-induced contractions by half, while no biologically relevant inhibitions occurred with lower concentrations or with carbachol- or methacholine-induced contractions. Pretensions before contractions were relaxed > 20% by 0.1–10 µM vibegron. EFS-induced contractions were halved by 10 µM vibegron in prostatectomized tissues, but unaffected by lower concentrations and in laser-enucleated prostate tissues. Additionally, 10 µM vibegron right-shifted concentration response curves and increased the EC_50_ values for noradrenaline, phenylephrine and methoxamine in prostatectomized and laser-enucleated prostate tissues, while lower concentrations had no consistent effect.

**Conclusions:**

Similar to mirabegron, improvements of storage symptoms by vibegron involve mechanisms beyond inhibition of voiding contractions. Off-target effects occur with 10 µM, and include inhibition of neurogenic contractions and antagonism of prostatic α_1A_-adrenoceptors. Vibegron effects in the prostate may differ between patients with low BPH progression, and patients needing surgery for BPH.

## Introduction

Vibegron is the second β_3_-adrenoceptor agonist approved for the treatment of storage symptoms in overactive bladder (OAB) (Michel et al. 2023; Nambiar et al. 2022). Its efficacy in symptom improvements is similar to mirabegron and to anticholinergics (Michel et al. 2023). In terms of tolerability, β_3_-adrenergic agonists appear to be superior to anticholinergics (Michel et al. 2023), so they could potentially replace them as the first-line option. Vibegron was approved based on results from clinical trials and animal studies, whereas, unlike mirabegron, no data from tests with human detrusor tissue were available at the time of approval and to date. Based on findings with mirabegron, mechanisms different from inhibition of voiding contractions in the detrusor may account for symptom improvements, and off-target effects in the prostate and bladder appear possible.

Based on observations of mirabegron-induced relaxation of isolated detrusor tissues, symptom improvements by mirabegron were initially explained by bladder smooth muscle relaxation or by inhibition of voiding contractions. Meanwhile, however, it emerged that these mechanisms are insufficient to explain its clinical effects (Huang et al. 2022c; Michel et al. 2025). Rather, neuronal β_3_-adrenoceptors in the central nervous system or spinal cord, or interference with microcontractions and the micturition reflex appear to be involved. Relaxations of precontracted human detrusor preparations are in fact small with physiological concentrations, and full muscarinic and neurogenic contractions of isolated human detrusor tissues are affected hardly, if at all even at concentrations exceeding the known plasma levels (Huang et al. 2022c). Nevertheless, the belief that β_3_-agonists improve storage symptoms by directly suppressing cholinergic voiding contractions through β_3_-adrenoceptors on bladder smooth muscle cells was widespread and is still circulating. In general, advances in drug development requires knowledge about effective mechansims of action, also of approved drugs. For the bladder and β-adrenergic ligands, studies in human tissues are finally particularly critical, as the subtype composition of β-adrenoceptor populations fundamentally differs between the human and non-human detrusor (Michel et al. 2023). Tissue studies with vibegron are currently limited to non-human bladders, where β-adrenoceptor populations can be composed by β_2_ and β_3_, whereas β_3_ is the major or single subtype in the humen detrusor (Michel et al. 2023).

In the prostate, α_1_-adrenoceptors are an important target for the treatment of voiding symptoms in benign prostatic hyperplasia (BPH) by α_1_-adrenergic antagonists (Gravas et al. 2023), and an off-target of mirabegron. Their off-target antagonism by mirabegron or other β_3_-adrenergic agonists could theoretically open up attractive possibilities for the dual treatment of storage and voiding symptoms (Alexandre et al. 2016; Calmasini et al. 2015; Huang et al. 2024). In human prostate tissues, however, antagonism of α_1_-adrenoceptors required mirabegron concentrations that do not occur in vivo (Huang et al. 2021). Findings with non-therapeutic concentrations in preclinical studies led to the assumption that mirabegron could improve voiding symptoms in BPH (Calmasini et al. 2015), which has not been confirmed in clinical studies. For vibegron, data for a possible antagonism of α_1_-adrenoceptors in the prostate are not yet available. If its affinity for α_1_-adrenoceptors is in ranges of plasma levels, improvements of voiding symptoms might be possible, so that preclinical investigations to that effect are of obvious clinical relevance. Storage and voiding symptoms often occur together in male patients. Their simultaneous treatment requires drug combinations, but polypharmacy is anyway highly prevalent in these patients (Gravas et al. 2023).

Affinity data, e.g. binding constants from radioligand assays, are apparently not available for vibegron, but EC_50_ values ​​for vibegron-induced cAMP production in cells transfected with β_3_-adrenoceptors (Alexander et al. 2023), suggesting that it has the highest known β_3_-selectivity from all clinically tested β_3_-agonists (Yamamoto et al. 2023). The EC_50_ for vibegron-induced cAMP production mounted to 1 nM (Di Salvo et al. 2017), 1.1 nM (increasing to 1.6 nM with 40% serum) (Edmondson et al. 2016), 1.3 nM (Yamamoto et al. 2023), or 2.1 nM (Brucker et al. 2022) with human β_3_-adrenoceptors, but > 10 µM (Di Salvo et al. 2017; Yamamoto et al. 2023), > 20 µM (Edmondson et al. 2016), or beyond detectable concentrations (Brucker et al. 2022) with human β_1_- and β_2_-adrenoceptors. Binding of vibegron to human α_1D_ or α_2B_ was lacking in competition assays with radiolabelled prazosin and rauwolscine (Brucker et al. 2022), but was observed in rat bladder tissues, where it inhibited binding of a muscarinic ligand, [N-methyl-^3^H]scopolamine with an IC_50_ of 1.52 µM (Yamada et al. 2021a). With a single standard dose of 75 mg, the peak plasma concentrations of vibegron amounted to 258 nM with intact pills and 182 nM with crushed pills (King et al. 2022), or to 299 nM (U.S. Food and Drug Administration 2019). Despite the clinical availability of vibegron, no data from human tissues are available that would provide information on effects on micturition contractions of the detrusor, or on concentration-effect relationships in the case of possible off-target effects in the prostate or bladder. Therefore, we here examined concentration-dependent effects of vibegron on contractions of human detrusor and prostate tissues.

## Materials and methods

### Human tissues, patients and structure of the study

Tissues were collected from three cohorts, including 1) detrusor tissues from radical cystectomy (rCx) for bladder cancer, 2) prostate tissues from radical prostatectomy (rPx) for prostate cancer but without prior surgery for BPH, and 3) prostate tissues from holmium and thulium laser enucleation of the prostate (HoLEP, ThuLEP) for BPH. In brief and as outlined in detail below, prostate tissues from laser enucleation represent advanced BPH but without prostate cancer, in patients with moderate or mostly severe and medication-refractory voiding symptoms, or with complications for BPH. In contrast, prostate tissues from rPx without prior surgery for BPH represent uncomplicated, non-surgical stages of BPH, i.e. from patients without or with only mild to moderate voiding symptoms. In contrast, no specific or general estimates are possible regarding the prevalence of OAB in our rCx cohort. This study was carried out in accordance with the Declaration of Helsinki of the World Medical Association and has been approved by the ethics committee of Ludwig Maximilian University, Munich, Germany. Written informed consent was obtained from all patients. All samples were collected and analyzed anonymously. All surgeries were performed at the Department of Urology of the LMU University Hospital. Cohorts subjected to rCx and rPx included exclusively tumor-positive patients. Hence, these surgeries were performed only for treatment of prostate or bladder cancer, while further conditions such as inflammation, OAB and others can not be excluded. Approximately 80% of rPx patients show BPH, but cancer patients with prior surgery for BPH (i.e., laser enucleation or transurethral resection of the prostate) were excluded from sampling for this study, because surgery for BPH results in ablation of the complete periurethral zone making the intended collection of this zone from rPx impossible (see below for details). Laser enucleation for BPH was exclusively performed for BPH, in patients with complications or at a high risk for complications, or with medication-refractory, strong symptoms, or who wish treatment but refuse medical treatment (Gravas et al. 2023), but without prostate cancer.

The total numbers of conducted experiments were 70 with detrusor tissues from radical cystectomy, 90 with prostate tissues from radical prostatectomy and 101 with prostate tissues from laser enucleation. However, precise numbers of involved patients can not be calculated due to anonymization after sampling, but ranged around 50 for tissues from radical cystectomy, 75 for tissues from radical prostatectomy and 80 for tissues from laser enucleation. The sizes of collected tissues varied, so that some samples were sufficient for only three organ bath channels in one experiment, but allowed two or more experiments in other cases (each with four channels). However, each tissue (i.e., a sample from a given patient) was only allocated once per series, so that, for example, n = 5 independent experiment in a series mean that tissues from n = 5 different patients were included.

In accordance with the anonymization, no patient-related data were collected, stored or evaluated. Detrusor tissues were collected from both female and male patients. The median age of patients cystectomized for bladder cancer at our department is 71.3 years, and 85% of the patients are male (December 2021 to March 2024, n = 154) (Ebner et al. 2025). The median age of patients undergoing prostatectomy for prostate cancer at our department is 66 years (n = 4,003, n = 5,800) (Grabbert et al. 2018; Westhofen et al. 2024b). In a cohort of 5,489 patients prostatectomized for prostate cancer at our department, 49.7% reported an international prostate symptom score (IPSS) ≥ 8 (defined as “LUTS in need of treatment”, averaging out to 14, age 67 years) and had a median prostate volume of 56 ml, while 50.3% had an IPSS < 8 (3 on average, 65 years) and a prostate volume of 49 ml (Westhofen et al. 2024a). In a cohort of 5,899 prostatectomized patients at our department, 99 patients received preoperative treatment with 5α-reductase inhibitors, reported a median IPSS of 7 and had a median prostate volume of 61.5 ml, while 5,800 did not take 5α-reductase inihibitors, had a median IPSS of 11 and a median prostate volume of 52 ml (Westhofen et al. 2024b). The prevalence of histological BPH in this age group (60–69 years) ranges between 60–70% (Lepor 2004), and the prevalence of “clinical BPH” (IPSS > 7, Q_max_ < 15 ml/s) up to 35% (Lepor 2004), whereas concomitant histological BPH is found in approximately 80% of prostate cancer patients (Alcaraz et al. 2009; Orsted and Bojesen 2013). In 1,593 patients undergoing HoLEP for LUTS due to benign prostatic obstruction (BPO) at our department (2018–2021), the median IPSS averaged out at 20, the Q_max_ at 10 ml/s, the postvoid residual urine volume (PVR) at 90, the prostate volume at 90 and the age at 71 years (Westhofen et al. 2023). In 606 propensity-matched patients again undergoing HoLEP for LUTS due to BPO at our department (2017–2020), the median IPSS was 21 in both groups (three-lobe and one-lobe enucleation, each n = 303), the Q_max_ 10 and 9 ml/s, the PVR 80 and 100 ml, and the age at 70 and 71 years (Tamalunas et al. 2023). In 852 age-stratified patients again treated by HoLEP for LUTS due to BPO at our department (2014–2018), the median IPSS ranged from 17–19, the PVR from 60–100, and the median Q_max_ amounted consistently to 11 ml/s in the three groups (PV ≤ 60 ml, > 60 and < 120 ml, ≥ 120 ml) (Tamalunas et al. 2022). According to the established cut-off points for IPSS-based staging of voiding symptoms, men scoring 0–7 points are considered mildly symptomatic, 8–19 points represent moderate symptoms, and 20–35 points reflect severe symptoms (Barry et al. 1992). Hence, patients undergoing rPx for prostate cancer show mostly mild or moderate symptoms, whereas symptoms are typically moderate to severe and associate with complications in laser-enucleated patients, reflecting advanced BPH.

### Human detrusor tissues from radical cystectomy

Detrusor tissues from the lateral bladder wall were dissected by a pathologist within 30–60 min after bladder removal, following longitudinal opening from the outlet to the bladder dome, and visual assessment of the bladder wall and intravesical surface for tumor infiltration. If the tumor burden permitted sampling, tissues were excised from non-infiltrated lateral areas, and the urothelium was removed. Experiments were initiated within 60 min of dissection. For intermediate storage and transport, organs and tissues were kept in Custodiol® solution (Köhler, Bensheim, Germany), with the bladder specimens being placed in containers containing Custodiol® immediately after removal in the operating room. Custodiol® is a cardioplegic and organ protective solution used in cardiac and other surgeries, and for transport and interim storage of organs for transplantation. Ready-to-use Custodiol® solution is commercially available, and contains 15 mM NaCl, 9 mM KCl, 4 mM MgCl_2_, 18 mM histidine-HCl, 180 mM free histidine (without HCl), 2 mM tryptophan, 30 mM mannitol, 0.015 mM CaCl_2_, and 1 mM potassium hydrogen-2-oxopentandioate (ketoglutarate), but no proteins, and has a pH between 6.92–7.3 (25 °C).

Radical cystectomy was performed exclusively for treatment of muscle invasive, and of highgrade non-muscle invasive bladder cancer. Eligibility criteria were age ≥ 18 years, and radical cystectomy for T2 or highgrade T1 bladder cancer. Exclusion criterion was a too high tumor burden on macroscopic examination, which precluded sample collection in order not to impair further diagnostics or because a reasonable sampling was no longer possible.

### Human prostate tissues from radical prostatectomy

Prostate tissues from the periurethral zone were dissected by a pathologist within 30–60 min after prostate removal, following opening by a longitudinal cut from the capsule to the urethra, and visual assessment of the intersections for tumor infiltration. Provided that tumor infiltration did not exclude sampling, tissues were cut from the periurethral zone, and organ bath experiments were initiated within 3 h after sampling. Macroscopically visible tumor infiltration in this region was rare (< 1% of prostates), consistent with the typical predominance of tumors in the peripheral zone (Pradidarcheep et al. 2011; Shaikhibrahim et al. 2012). For intermediate storage and transport, organs and tissues were kept in Custodiol® solution, with the prostate specimens being placed in containers containing Custodiol® immediately after removal in the operating room.

Radical prostatectomy was performed exclusively for treatment of prostate cancer. In general, hormone or chemotherapy is reserved for palliative settings or metastatic prostate cancer. Consequently, none of the patients included in our cohort had received systemic treatment before undergoing prostatectomy. Eligibility criteria were age ≥ 18 years, male sex assigned at birth, and radical prostatectomy for localized prostate cancer (≤ T2). Exclusion criteria were prior surgery for lower urinary tract symptoms/BPH, salvage prostatectomy, locally advanced prostate cancer (T3–4), previous chemotherapy, radiotherapy, or hormone therapy. Prostates from individuals with a prior BPH surgery were excluded from tissue collection, as this procedure removes the periurethral zone entirely.

### Human prostate tissues from laser enucleation

HoLEP and ThuLEP were carried out using a three-lobe enucleation approach, as recently detailed (Keller et al. 2025a). After retrieval of the morcellated tissue fragments from the bladder, samples were promptly placed in Custodiol® solution still in the operation room, for transport and interim storage, and during selection of shreds for subsequent use in organ bath experiments. Experiments were initiated within 2 h of tissue extraction.

According to guidelines of the European Association of Urology (EAU), surgery for BPH is required in patients with recurrent or refractory urinary retention, overflow incontinence, recurrent infections of the urinary tract infections, diverticula or bladder stones, treatment-resistant macroscopic haematuria due to BPH, or dilatation of the upper urinary tract due to benign prostatic obstruction with or without renal insufficiency. In addition to these absolute operation indications (need for surgery), surgery is recommended with relative surgical indications, i.e. for patients showing inadequate relief from symptoms or of postvoid residual urine by conservative or medical treatment. Finally, surgery is performed in patients with severe symptoms, who wish treatment but refuse medical options. In fact, decisions for desobstructive surgery real world settings are often based on routine care for male LUTS, and do not always include diagnosis by invasive urodynamics (Drake et al. 2017). An estimated 18–28% of patients undergoing prostate surgery for LUTS have no obstruction (Young et al. 2017). Inclusion criteria in our study were age ≥ 18 years, male sex assigned at birth, bladder outlet obstruction, and intervention by HoLEP or ThuLEP. Exclusion criteria were prostate cancer, and intervention by transurethral resection of the prostate (TURP) or by other surgical approaches for treatment of BPH (except HoLEP or ThuLEP).

### Organ bath

Strips with sizes about 6 × 3 × 3 mm were cut from tissues sampled from rCx or rPx, and collected without further cutting or prepared from the largest shreds from laser-enucleated tissues. Tissue strips were placed in four-channel myograph systems (Model 720 M; Danish Myotechnology, Aarhus, Denmark), with each chamber containing 10 ml of Krebs–Henseleit solution maintained at 37 °C and continuously aerated with carbogen (95% O_2_, 5% CO_2_). After a stable pretension of 4.9 mN was adjusted within 45 min (Huang et al. 2021; Huang et al. 2022c), contractions were induced by 80 mM KCl. After the maximum plateau contraction was reached, the solution in the chambers was washed out, resulting in a new baseline. Subsequently, vibegron in indicated concentrations was added to two chambers of a device, or dimethylsulfoxide (DMSO, used as solvent for vibegron) for controls to the two other chambers, with all four chambers filled with tissue from the same organ, resulting in paired samples in the control and vibegron groups. After further 30 min, concentration response curves for agonists or frequency response curves for electric field stimulation (EFS) were constructed. EFS was performed as previously described (Huang et al. 2021, 2022c), and only one curve was recorded per sample. The allocation of tissues to the vibegron or control group was done intuitively, i.e., this was changed between experiments.

Each individual experiment contained a vibegron and a vehicle group, with tissues in both groups being obtained from the same organ. Each group per experiment was determined as double determination, wherever this was possible (i.e., two channels with vibegron, two channels for control). Double determinations in both groups were performed in 61 of 70 experiments with detrusor tissues, in 78 of 90 experiments with prostate tissue from rPx and in 55 of 101 experiments with prostate tissues from laser enucleation. In the remaining experiments, tissue availability was insufficient to fill two channels per group, or samples failed to respond to KCl stimulation. In such cases, single measurements were conducted in one of the two groups (8 experiments with detrusor tissue, 12 experiments with prostate tissue from rPx, 26 experiments with laser-enucleated prostate tissue), or single determinations in both groups were performed (1 experiment with detrusor tissue, 20 experiments with laser-enucleated prostate tissue).

Contractile responses to agonists and EFS were normalized to the reference contraction induced by 80 mM KCl, to compensate for inter-sample variability including differences in strip dimensions, smooth muscle content, or any other heterogeneity. E_max_ and EC_50_ values for agonist-induced responses, as well as Ef_50_ values (frequency inducing half-maximal EFS contraction), were determined individually for each experiment by non-linear curve fitting (Huang et al. 2022a), using GraphPad Prism 6 (GraphPad Software Inc., San Diego, CA, USA). The program displays error messages if curve fitting fails, or marks the results as "ambiguous" if they appear ambiguous or implausible. Following the manufacturer's recommendations ("GraphPad Curve Fitting Guide", GraphPad Software Inc.), the results were also manually checked for plausibility. Error messages or implausible results occur most frequently in experiments in which the concentration–response curves show pronounced "downhill" parts, which can often occur at higher agonist concentrations. Consequently, each curve was manually checked, and only parts including the concentrations before the curve maximum was reached were used for analysis if curve fitting was problematic due to sustained downhill parts. This applied to most of the agonist experiments performed with detrusor tissues, to two experiments with noradrenaline (control groups for 100 nM vibegron) with laser-enucleated tissues, but to only one experiment with prostate tissues from radical prostatectomy (performed with U46619). Curve fitting and exclusion of downhill parts was impossible in the control group of one experiment (carbachol-induced contractions, detrusor tissues, 1 µM vibegron). Consequently, the highest tension in this curve was taken as the corresponding E_max_ value, and an EC_50_ of −7 was estimated for analysis of curve fitting data, with these values ​​marked in gray in the scatter plot.

To estimate the affinity of vibegron to muscarinic receptors and α_1_-adrenoceptors, “apparent” pA_2_ values were determined as an approximation to true pA_2_ values. Values were calculated separately for all independent experiments, as the sum of the negative decadic logarithm of the vibegron concentration (5, as increases in EC_50_ values were only observed with 10 µM vibegron), and the right shift in concentration response curves for α_1_-adrenergic or muscarinic agonists, expressed as negative decadic logarithm: apparent pA_2_ = p[vibegron] + (pEC_50_ α_1_/muscarinic agonist controls – pEC_50_ α_1_/muscarinic agonist with vibegron). Our study design and the resulting data structure do not allow the calculation of true pA_2_ values, as this requires determinations with multiple ligand concentrations compared to shared control group in the same experiment.

### Materials, drugs and nomenclature

Vibegron ((6S)-N-[4-[[(2S,5R)−5-[(R)-hydroxy(phenyl)methyl]pyrrolidin-2-yl]methyl]phenyl]−4-oxo-7,8-dihydro-6H-pyrrolo[1,2-a]pyrimidine-6-carboxamide) is a β_3_-adrenoceptor agonist (Alexander et al. 2023), which is available for treatment of OAB (Michel et al. 2023), and was purchased from MedChemExpress (Monmouth Junction, NJ, USA). Stock solutions (1000-fold of final concentrations) were prepared in DMSO and stored as small aliquots at −20 °C until use. Phenylephrine ((R)−3-[−1-hydroxy-2-(methylamino)ethyl]phenol) and methoxamine (α-(1-Aminoethyl)−2,5-dimethoxybenzyl alcohol) are α_1_-selective adrenoceptor agonists. U46619 ((Z)−7-[(1R,4S,5S,6R)−6-[(E,3S)−3-hydroxyoct-1-enyl]−2-oxabicyclo[2.2.1]heptan-5-yl]hept-5-enoic acid) is an agonist for the thromboxane A_2_ (TP) receptor. Carbachol (2-carbamoyloxyethyl(trimethyl)azanium) and methacholine (2-acetyloxypropyl(trimethyl)azanium) are agonists for muscarinic acetylcholine receptors. Stock solutions (10 mM) of noradrenaline, phenylephrine, methoxamine, carbachol and methacholine were freshly prepared in deionized water before each experiment. Stock solutions of U46619 (10 mM) were prepared in ethanol as small aliquots at −80 °C until use. Stock solutions of endothelin-1 (0.4 mM) were prepared in DMSO, and stored as small aliquots at −20 °C until use. Noradrenaline, phenylephrine, methoxamine, carbachol and methacholine were obtained from Sigma-Aldrich (Munich, Germany). U46619 and endothelin-1 were obtained from Enzo Life Sciences (Lörrach, Germany). DMSO, KCl and all other chemicals required for the preparation of Krebs–Henseleit solution were obtained from Carl Roth (Karlsruhe, Germany).

### Statistical analyses

Data are presented as means ± standard deviation (SD) in concentration and frequence response curves, and as individual values from each experiment together with means in scatter plots for EC_50_ EF_50_ and E_max_ values. In the text, data are reported as means with 95% confidence intervals (95% CIs). The data in each data set, i.e., in each diagram are paired, as both groups per experiment were formed by tissue from the same patient. Statistical analyses, normality tests and calculation of 95% CIs were performed using GraphPad Prism 6. Frequence and concentration response curves were analyzed by two-way analysis of variance (ANOVA) as recently described (Huang et al. 2022a), without testing for normality due to the absence of non-parametric alternatives for 2-way ANOVA. Paired data sets containing E_max_, EF_50_ and EC_50_ values with ≥ 7 values per group were assessed for normality using the Shapiro–Wilk normality test, and compared by paired Student’s t-test if values in both groups showed normal distribution, or by Wilcoxon matched-pairs signed rank test if non-Gaussian distribution was observed in at least one of both groups. Groups including n = 5–6 values from curve fitting were underpowered for valid analysis by D'Agostino & Pearson omnibus normality or Shapiro–Wilk normality tests, as these are not supported in GraphPad Prism 6 with group sizes < 7. Consequently, distribution in these data sets was visually inspected, and groups were analyzed by paired Student’s t-test if a normal distribution could be reasonably assumed, or by Wilcoxon matched-pairs signed rank test if a normal distribution could be confidently excluded in at least one group. Precise information about which test was applied to which data sets is provided in the figure legends. P values < 0.05 were considered significant. The present study and analyses show an exploratory design, as typical features of a strictly hypothesis-testing study were lacking, including blinding, biometric calculation of group sizes, or adherence to predefined group sizes (Michel et al. 2020). Consequently, and aligning with the limitations associated to normality testing of our data, p values reported are descriptive, but not hypothesis-testing (Michel et al. 2020). Interpretation of results was based on effect sizes and their possible relevance, instead of p values.

Group sizes were initially preplanned to n = 5 per experimental series, i.e., n = 5 independent experiments, with tissues from five different patients, with both groups per experiment derived from the same individual. This number was exceeded in some series (up to n = 8), due to staff transitions and the involvement of three different investigators performing organ bath experiments at different times, which led to overlapping contributions and parallel continuation of these same series during handovers. Unlike our previous studies, however, group sizes were not increased based on the results of statistical tests in interim analyses of five initial experiments.

No data were omitted from analyses, apart from downhill parts in concentration response curves during curve fitting of three experiments, as described above. Two experiments were omitted because the contractions were obviously completely outside the variation, including one with 10 nM vibegron and noradrenaline in HoLEP tissues (noradrenaline-induced tensions from −38% to −17% of KCl in control group, −35% to 13% with vibegron) and one with 100 nM vibegron and EFS in HoLEP tissues (EFS-induced tensions from −7% to 4% of KCl in control group, −7% to −10% with vibegron).

## Results

### Contractions by muscarinic agonists in detrusor tissues from radical cystectomy

**Fig. 1:**
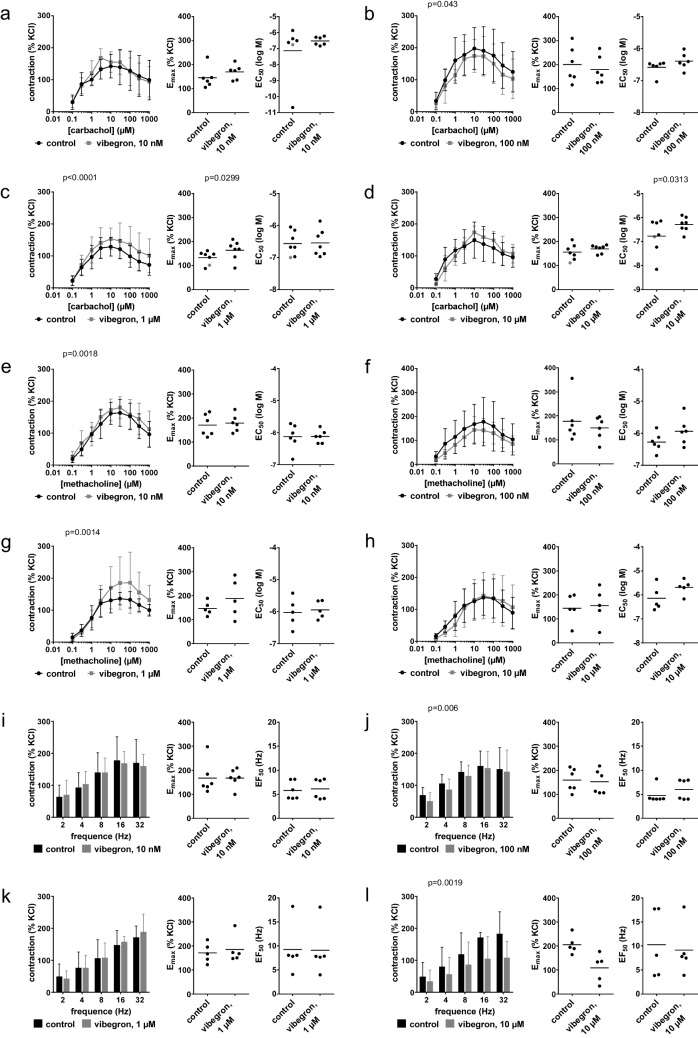
Effects of vibegron on contractions by muscarinic agonists and EFS in human detrusor tissues from radical cystectomy for bladder cancer. Concentration or frequency response curves were constructed for carbachol **(a-d)**, methacholine **(e–h)** and noradrenaline **(i-l)**, in the presence of 10 nM (a, e, i), 100 nM (b, f, j), 1 µM (c, g, k) or 10 µM (d, h, l) vibegron, or solvent (controls). Data are from 5 (g, h, k, l), 6 (a, b, e, f, i, j) or 7 independent experiments (c, d) per diagram, where tissues from 5 (g, h, k, l), 6 (a, b, e, f, i, j) or 7 (c, d) patients were distributed to the vibegron and control group of a panel. Data are means ± standard deviation from all experiments in concentration frequency response curves, shown together with p values from two-way ANOVA, and all individual E_max_ and EC_50_ values from each experiment in scatter plots together with means and p values from paired Student’s t-tests (applied in b (E_max_), c, d (E_max_), e (E_max_), f (EC_50_), g, h (EC_50_), i (EF_50_), j (E_max_), l (E_max_)) or Wilcoxon matched-pairs signed rank test (applied in a, b (EC_50_), d (EC_50_), e (EC_50_), f (E_max_), h (E_max_), i (E_max_), j (EF_50_), k, l (EF_50_)). An E_max_ and EC_50_ value marked in grey represent the maximum tension in the concentration response curves and an estimated EC_50_ value, as curve fitting was not possible in the control group of this experiment

Effects of vibegron on carbachol-induced (Fig. 1a-d) and methacholine-induced contractions (Fig. 1e-h) suggested no biological relevant attenuations of cholinergic contractions in detrusor tissues. Decreases with 100 nM vibegron were minor, but seen in concentration response curves for both agonists and reflected by E_max_ values, amounting to 201% of KCl-induced contraction [122 to 279] in controls and 179% [119 to 240] with vibegron for carbachol (Fig. 1b), and 177% [81 to 274] in controls and 150% [99 to 201] with vibegron for methacholine (Fig. 1f). In contrast, increased contractions were seen with 1 µM vibegron in concentration response curves for both agonists and mirrored by E_max_ values, amounting to 133% [107 to 158] in controls and 164% [127 to 201] with vibegron for carbachol (Fig. 1c), and 147% [112 to 183] in controls and 189% [88 to 289] with vibegron for methacholine (Fig. 1g). At 10 µM vibegron, no consistent effects were observed in concentration response curves or on E_max_ values, but EC_50_ values (log M) were increased from −6.8 [−7.5 to −6.1] in controls to −6.3 [−6.6 to −6] with vibegron for carbachol (Fig. 1d), and from −6.1 [−6.8 to −5.5] in controls to −5.7 [−6.1 to −5.3] with 10 µM vibegron for methacholine (Fig. 1h).

### EFS-induced contractions in detrusor tissues from radical cystectomy

EFS-induced contractions were unchanged with 10 nM (Fig. 1i), 100 nM (Fig. 1j) and 1 µM vibegron (Fig. 1k), but reduced with 10 µM vibegron (Fig. 1l). E_max_ values for EFS-induced contractions amounted to 205% of KCl-induced contractions [158 to 253] in controls and 109% [36 to 181] with 10 µM vibegron (Fig. 1l).

### Vibegron-induced relaxations of pretensions in detrusor tissues from radical cystectomy

**Fig. 2:**
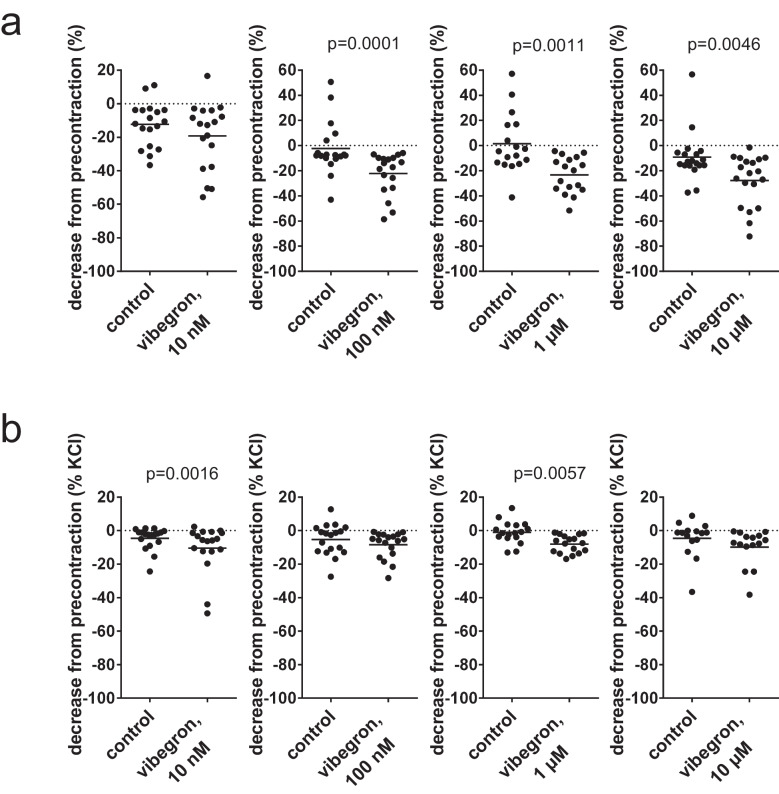
Relaxation of human detrusor tissues from radical cystectomy for bladder cancer by vibegron. Changes in pretensions (approximately 4.9 mN, after washout of highmolar KCl) within 30 min after addition of solvent or vibegron before construction of concentration or frequence response curves (Fig. 5) were calculated as % decrease, by refering the pretension 30 min after vibegron or solvent addition to the pretension before addition (i.e., after washout of KCl) **(a)**, and by normalizing the decreases in pretension to the KCl-induced contractions **(b)**. Shown are all single values from each experiment together with means from paired Student’s t-test (applied to 10 nM and 1 µM in (a), and to 1 µM in (b)) or Wilcoxon matched-pairs signed rank test (applied to 100 nM and 10 µM in (a), and to 10 nM, 100 nM and 10 µM in (b))

Changes in pretension after addition of vibegron or solvent were calculated both as a % decrease, by refering the pretension 30 min after vibegron or solvent addition to the pretension before addition (i.e., after washout of KCl) (Fig. 2a), and by normalizing the decreases in pretension to the KCl-induced contractions (Fig. 2b). If refererred to pretensions before addition, changes in pretension were similar between solvent and 10 nM vibegron, but possible relaxations in pretension occurred with 100 nM, 1 µM and 10 µM vibegron (Fig. 2a). Changes in pretensions amounted to −19% [−29 to −9] with 10 nM vibegron and −12% [−19 to −6] in controls, −22% [−30 to −14] with 100 nM and −2% [−13 to 8] in controls, −23% [−31 to −16] with 1 µM and 1% [−11 to 14] in controls, and −28% [−37 to −18] with 10 µM and −9% [−19 to 0] in controls (Fig. 2a). Referred to highmolar KCl-induced contractions, changes in pretensions amounted to −10% [−18 to −3] with 10 nM vibegron and −5% [−8 to −1] in controls, −9% [−12 to −5] with 100 nM and −5% [−10 to −1] in controls, −8% [−11 to −3] with 1 µM and −1% [−4 to 2], and −10% [−16 to −4] with 10 µM and −5% [−11 to 1] in controls (Fig. 2b).

### Contractions by α_1_-adrenergic agonists in prostate tissues from radical prostatectomy

**Fig. 3:**
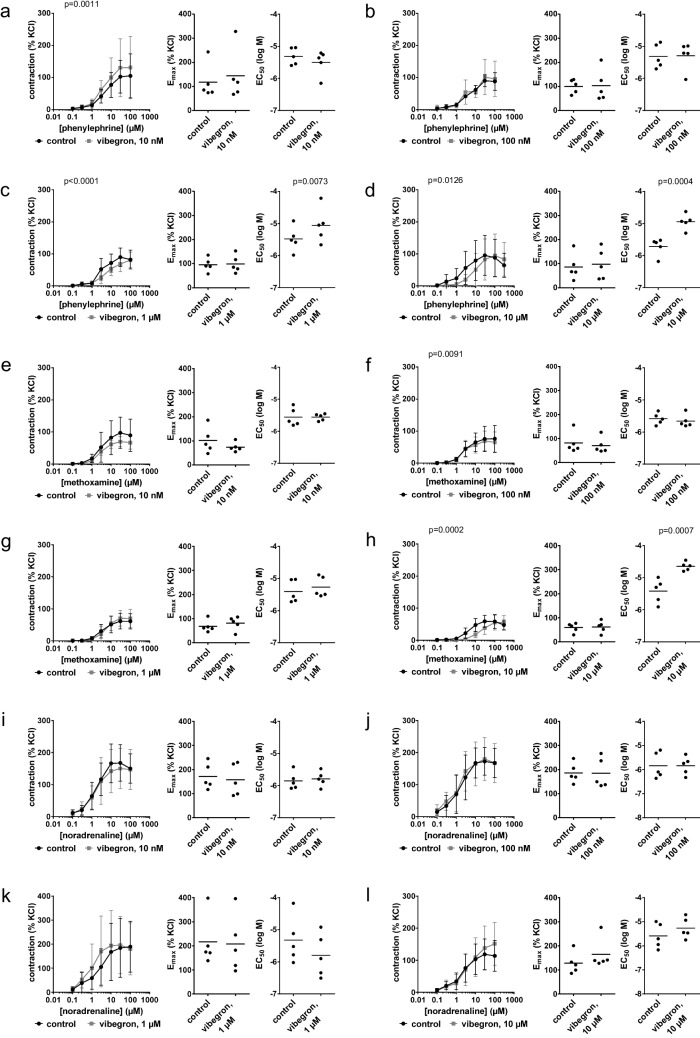
Effects of vibegron on contractions by α_1_-adrenergic agonists in human prostate tissues from radical prostatectomy for prostate cancer. Concentration response curves were constructed for phenylephrine **(a-d)**, methoxamine **(e–h)** and noradrenaline **(i-l)**, in the presence of 10 nM (a, e, i), 100 nM (b, f, j), 1 µM (c, g, k) or 10 µM (d, h, l) vibegron, or solvent (controls). Data are from 5 independent experiments per diagram, where tissues from 5 patients were distributed to the vibegron and control group of a panel. Data are means ± standard deviation from all experiments in concentration response curves, shown together with p values from two-way ANOVA, and all individual E_max_ and EC_50_ values from each experiment (calculated by curve fitting) in scatter plots together with means and p values from paired Student’s t-tests (applied in c, d, e, f, g, h, i, j) or Wilcoxon matched-pairs signed rank test (applied in a, b, k, l)

Vibegron at a concentration of 10 µM right shifted the concentration response curves for phenylphrine and methoxamine and increased the EC_50_ values for both agonists, while no other consistent effects were observed (Fig. 3). Phenylephrine-induced contactions were not attenuated by either 10 nM (Fig. 3a) or 100 nM (Fig. 3b) vibegron, but right shifted by 1 µM (Fig. 3c) and 10 µM (Fig. 3d) vibegron. Right shifts included full recovery of maximum contractions at high phenylephrine concentrations, and were reflected by increased EC_50_ values for phenylephrine with 1 µM and 10 µM vibegron (Fig. 3c, d). The EC_50_ values for phenylephrine (log M) were −5.5 [−6 to −5] in controls and −5.1 [−5.7 to −4.4] with 1 µM vibegron (Fig. 3c), and −5.7 [−6.1 to −5.4] in controls and −4.9 [−5.2 to −4.6] with 10 µM vibegron (Fig. 3d).

Methoxamine-induced contractions were reduced slightly, though insignificantly by 10 nM vibegron (Fig. 3e), but unchanged at 100 nM (Fig. 3f) and 1 µM vibegron (Fig. 3g). Concentration response curves for methoxamine were right shifted by 10 µM vibegron, with full recovery at high methoxamine concentrations and reflected by increased EC_50_ values for methoxamine (Fig. 3h). The EC_50_ value for methoxamine (log M) was −5.4 [−5.9 to 5] in controls and −4.6 [−4.8 to −4.5] with 10 µM vibegron (Fig. 3h).

Noradrenaline-induced contrations were not attenuated with 10 nM (Fig. 3i), 100 nM (Fig. 3j), 1 µM (Fig. 3k) or 10 µM vibegron (Fig. 3l). A left shift of concentration response curves with 1 µM, with EC_50_ values for noradrenaline (log M) of −5.3 [−6.2 to −4.4] in controls and −5.8 [−6.6 to −5] with 1 µM vibegron (Fig. 3k), and an increase in the E_max_ for noradrenaline from 128% of KCl-induced contractions [72 to 183] in controls to 164% [86 to 242] with 10 µM vibegron (Fig. 3l) were neither consistent with phenylephrine or methoxamine, nor with other vibegron concentrations applied to noradrenaline.

### EFS-induced contractions in prostate tissues from radical prostatectomy

**Fig. 4:**
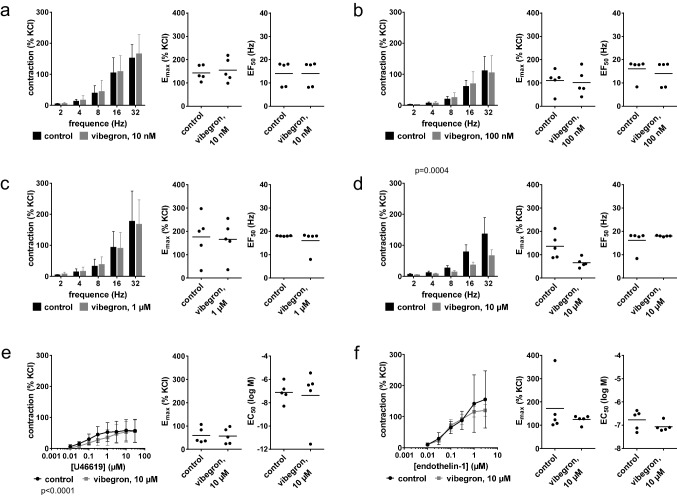
Effects of vibegron on contractions by EFS and non-adrenergic agonists in human prostate tissues from radical prostatectomy for prostate cancer. Frequency or concentration response curves were constructed for EFS **(a-d)**, U46619 **(e)** and endothelin-1 **(f)**, in the presence of 10 nM (a), 100 nM (b), 1 µM (c) or 10 µM (d-f) vibegron, or solvent (controls). Data are from 5 independent experiments per diagram, where tissues from 5 patients were distributed to the vibegron and control group of a panel. Data are means ± standard deviation from all experiments in concentration and frequency response curves, shown together with p values from two-way ANOVA, and all individual E_max_ and EC_50_ values from each experiment (calculated by curve fitting) together with means in scatter plots analyzed by paired Student’s t-test (a, d (E_max_), e (E_max_), f (EC_50_) or Wilcoxon matched-pairs signed rank test (b, c, d (EC_50_), e (EC_50_), f (E_max_))

EFS-induced contractions were unchanged with 10 nM (Fig. 4a), 100 nM (Fig. 4b) and 1 µM vibegron (Fig. 4c), but reduced with 10 µM vibegron (Fig. 4d). The E_max_ for EFS-induced contractions amounted to 136% of KCl-induced contractions [71 to 202] in controls and 66% [40 to 91] with 10 µM vibegron (Fig. 4d).

### Non-adrenergic contractions in prostate tissues from radical prostatectomy

Contractions by U46619 (Fig. 4e) and endothelin-1 (Fig. 4f) were affected only marginally if at all, likely without biological relevance.

### Contractions by α_1_-adrenergic agonists in prostate tissues from laser enucleation

**Fig. 5:**
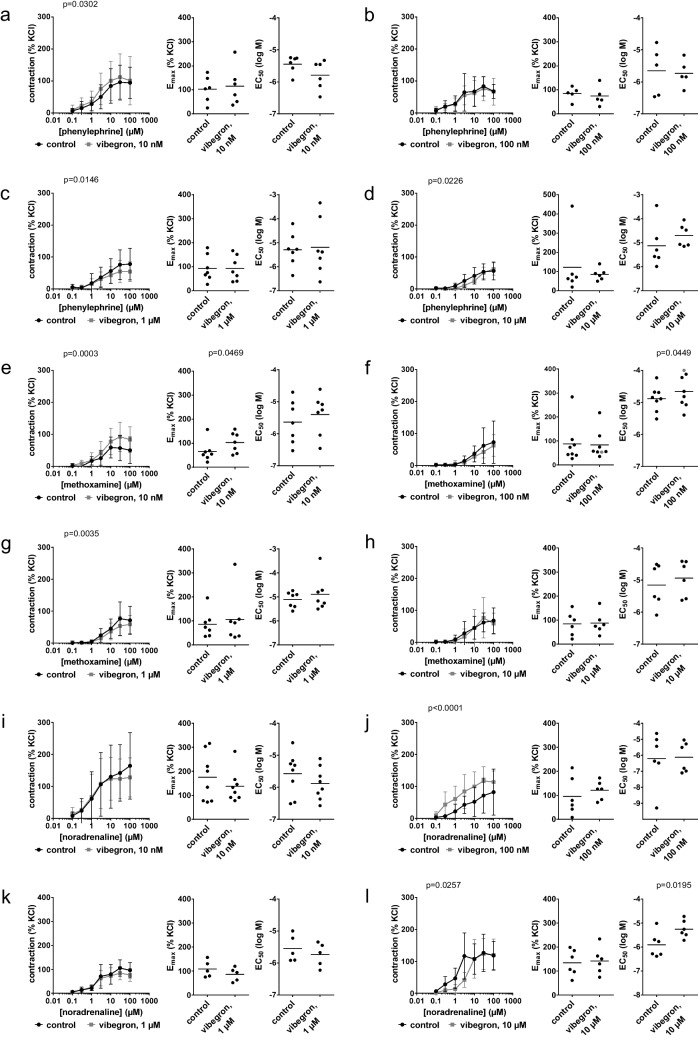
Effects of vibegron on contractions by α_1_-adrenergic agonists in human prostate tissues from laser enucleation for BPH. Concentration response curves were constructed for phenylephrine **(a-d)**, methoxamine **(e–h)** and noradrenaline **(i-l)**, in the presence of 10 nM (a, e, i), 100 nM (b, f, j), 1 µM (c, g, k) or 10 µM (d, h, l) vibegron, or solvent (controls). Data are from 5 (b, k), 6 (a, d, h, j, l), 7 (c, e, g) or 8 independent experiments (f, i) per diagram, where tissues from 5 (b, k), 6 (a, d, h, j, l), 7 (c, e, g) or 8 (f, i) patients were distributed to the vibegron and control group of a panel. Data are means ± standard deviation from all experiments in concentration response curves, shown together with p values from two-way ANOVA, and all individual E_max_ and EC_50_ values from each experiment (calculated by curve fitting) in scatter plots together with means and p values from paired Student’s t-tests (applied in a, b, c, e (EC_50_), f (EC_50_), h, i (EC_50_), j (E_max_), k, l) or Wilcoxon matched-pairs signed rank test (applied in d, e (E_max_), f (E_max_), g, i (E_max_), j (EC_50_))

Contractions by phenylephrine (Fig. 5a-d) and methoxamine (Fig. 5e-h) were not consistently altered by 10 nM, 100 nM, 1 µM or 10 µM vibegron. An increase in E_max_ values for methoxamine from 65% of KCl-induced contractions [25 to 106] in controls to 103% [64 to 143] with 10 nM vibegron (Fig. 5e) was partly mirrored by minor increases in concentration response curves for phenylephrine (Fig. 5a), but not observed with noradrenaline (Fig. 5i). Minor decreases in concentration response curves for phenylephrine with 1 µM (Fig. 5c) and 10 µM vibegron (Fig. 5d) were reflected by increases in the EC_50_ (log M) for phenylephrine from to −5.1 [−6.1 to −4.2] in controls to −4.7 [−5.2 to −4.2] with 10 µM vibegron (Fig. 5d), but not by any other parameters calculated by curve fitting. Changes in EC_50_ values were limited to a small increase from −4.9 [−5.2 to −4.6] in controls to −4.7 [−5.1 to −4.2] with 100 nM vibegron for methoxamine (Fig. 5e-h), while no changes were observed with 10 nM (Fig. 5i), 100 nM (Fig. 5j) or 1 µM vibegron (Fig. 5k) for noradrenaline. A possible right shift in concentration response curves for noradrenaline by 10 µM vibegron was paralleled by an increase in the EC_50_ for noradrenaline from −5.9 [−6.5 to −5.4] in controls to −5.3 [−5.6 to −4.9] with 10 µM vibegron (Fig. 5l).

### EFS-induced contractions in prostate tissues from laser enucleation

**Fig. 6:**
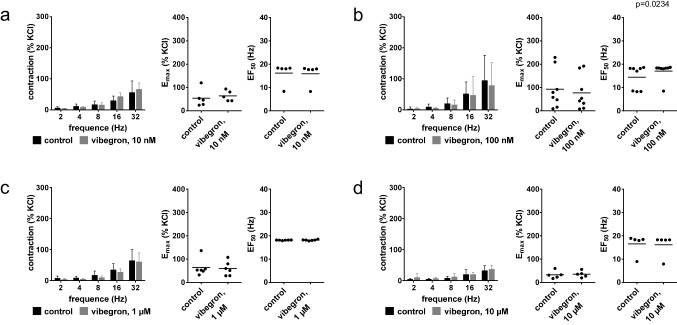
Effects of vibegron on contractions by EFS in human prostate tissues from laser enucleation for BPH. Frequency response curves were constructed for EFS, in the presence of 10 nM **(a)**, 100 nM **(b)**, 1 µM **(c)** or 10 µM **(d)** vibegron, or solvent (controls). Data are from 5 (a, d), 6 (c) or 8 independent experiments (b) per diagram, where tissues from 5 (a, d), 6 (c) or 8 (b) patients were distributed to the vibegron and control group of a panel. Data are means ± standard deviation from all experiments in frequency response curves (analyzed by two-way ANOVA), and all individual E_max_ and EC_50_ values from each experiment (calculated by curve fitting) in scatter plots together with means and p value from paired Student’s t-tests or Wilcoxon matched-pairs signed rank test (applied to all data in scatter plots except EF_50_ values in (c) and E_max_ values in (d), which were analyzed by Student’s t-test)

EFS-induced contractions were unchanged by 10 nM (Fig. 6a), 100 nM (Fig. 6b), 1 µM (Fig. 6c) and 10 µM vibegron (Fig. 6d).

### Apparent pA_2_values

**Fig. 7:**
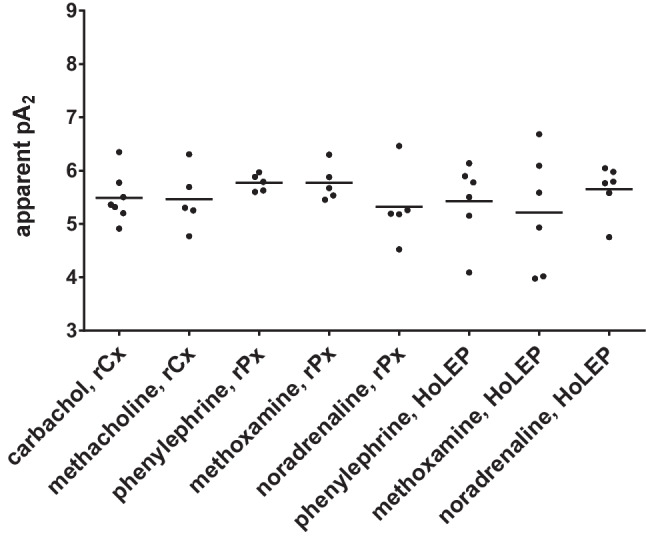
Apparent pA_2_ values of vibegron. Apparent pA_2_ values were calculated for series with 10 µM verapamil, applied to carbachol and methoxamine in experiments with detrusor tissues, and to phenylephrine, methoxamine and noradrenaline in experiments with prostate tissues from rPx and HoLEP. Calculation was based on experiments shown in Figs. 1, 3 and 5. Shown are all values together with standard deviation from all single, independent experiments

Apparent pA_2_ values were calculated for series addressing effects of 10 µM vibegron on contractions by muscarinic agonists in detrusor tissues and by α_1_-adrenergic contractions in prostate tissues (Fig. 7). Apparent pA_2_ values amounted to 5.49 [5.06 to 5.92] with carbachol, 5.47 [4.75 to 6.18] with methacholine, 5.78 [5.58 to 5.97] with phenylephrine in rPx tissues, 5.77 [5.35 to 6.19] with methoxamine in rPx tissues, 5.32 [4.45 to 6.2] with noradrenaline in rPx tissues, 5.43 [4.65 to 6.2] with phenylephrine in HoLEP tissues, 5.21 [4.05 to 6.37] with methoxamine in HoLEP tissues, and 5.65 [5.16 to 6.14] with noradrenaline in HoLEP tissues.

## Discussion

Our findings support the concept that β_3_-adrenergic agonists improve storage symptoms independently from inhibitions of voiding contractions in the detrusor, and suggest off-target effects of vibegron on human bladder and prostate smooth muscle contraction. At concentrations corresponding to plasma levels, attenuations of agonist-induced and neurogenic contractions by vibegron were lacking or small in detrusor and prostate tissues, so that improvements of storage or voiding contractions through smooth muscle contraction inhibition are unlikely. However, relaxations of pretensions seen with nanomolar concentrations may not preclude inhibitions of microcontractions inducing the voiding reflex in the micturition cycle and in OAB. Off-target effects include antagonism of muscarinic receptors in the detrusor and of α_1_-adrenoceptors in the prostate, but required concentrations probably exceeding the plasma levels of vibegron, so that these are unlikely to occur in vivo. Our findings with vibegron are similar to those with mirabegron obtained under similar conditions (Huang et al. 2021, 2022c), suggesting that these features may be at least partly class effects of β_3_-adrenergic agonists. Thus, separation of target and off-target effects appears important in preclinical studies with β_3_-agonists, as findings with supra-therapeutic concentrations in preclinical studies are of limited clinical relevance, but led to false conclusions in the past.

With a single standard dose of 75 mg, the peak plasma levels of vibegron amount to 182–299 nM (King et al. 2022; U.S. Food and Drug Administration 2019). The EC_50_ values for vibegron-induced cAMP production in β_3_-adrenoceptor-transfected cell culture models mounted to 1–2.1 nM (Brucker et al. 2022; Di Salvo et al. 2017; Edmondson et al. 2016; Yamamoto et al. 2023), whereas affinity data from competition and binding assays are not available. For the concentrations used in our study, this means, firstly, that any existing β_3_-adrenergic effect could be expected at 10 nM, and secondly, that any effect seen with 100 nM could also be expected in vivo, whereas plasma levels of 1 µM hardly and of 10 µM definitely do not occur in vivo. Bladder emptying in voiding is caused by muscarinic detrusor contractions (Michel et al. 2023). However, carbachol-, methacholine- or EFS-induced contractions were not reduced or only to smallest (possibly biologically not relevant) extent by 10 nM, 100 nM or 1 µM vibegron in our experiments with detrusor tissues. Hence, it appears unlikely that vibegron improves storage symptoms by inhibitions of voiding contractions.

Consequently, increases in EC_50_ values by 10 µM vibegron that were observed with carbachol and methacholine may represent off-target effects, probably by antagonism of muscarinic receptors and possibly accounting for the inhibition of EFS-induced contractions at 10 µM. The affinity of 3.4–4.7 µM for muscarinic receptors estimated on the basis of our apparent pA_2_ values may reflect binding to M_3_ receptors, as this subtype mediates muscarinic contractions in human bladder smooth muscle (Michel et al. 2023). In contrast, the previously reported IC_50_ of 1.52 µM for competition of a muscarinic ligand by vibegron in rat bladder tissues may reflect mixed binding to M_2_ and M_3_ receptors (Yamada et al. 2021a). The functional role of M_2_ receptors in the bladder remains to be fully understood (Michel et al. 2023). Antagonism of muscarinic receptors has been suggested as well for mirabegron, which replaced a muscarinic ligand in rat bladder tissues with an IC_50_ of 2.4 µM (Yamada et al. 2021b). Unlike vibegron, 10 µM mirabegron slightly increased EC_50_ values for methacholine but not carbachol in contraction of human detrusor tissues (Huang et al. 2022c), together suggesting a lower off-target affinity of mirabegron to M_3_ receptors compared to vibegron.

With therapeutic plasma concentrations, direct binding of vibegron to muscarinic receptors appears unlikely in vivo. However, urine concentrations may be higher than plasma concentrations, and have been extrapolated to 1.6–16 µM (Yamada et al. 2021a). Assuming that the affinity of vibegron to muscarinic receptors is the same in rat and human bladder, it was supposed that these urine concentrations result in an occupancy of 51–91% of the muscarinic receptor population in the human bladder (Yamada et al. 2021a). However, these values are extrapolations. If measurements confirm these intravesical vibegron concentrations, the question remains whether penetration across the urothelium, exerting an important barrier function in the bladder is sufficient to reach micromolar concentrations within the detrusor tissue. Unlike rat tissues, where the affinity of vibegron to muscarinic receptor populations was determined to 1.52 µM (Yamada et al. 2021a), cholinergic contractions were affected only to small degree up to 10 µM vibegron in our in vitro experiments, while an inhibition of EFS-induced contractions (possibly by inhibition of cholinergic neurotransmission) occurred with 10 µM. Micromolar concentrations can not be excluded with full confidence in the bladder wall, but can not be expected in the prostate, so that the antagonism of α_1_-adrenergic contractions by 10 µM is highly unlikely to occur in vivo. In general, supra-therapeutic drug concentrations are often applied in preclinical studies, which may lead to premature conclusions. Based on inhibitions of α_1_-adrenergic prostate smooth muscle contractions using 1 µM and 10 µM mirabegron, it was speculated that mirabegron may improve voiding symptoms (Calmasini et al. 2015). However, mirabegron did not improve urinary flow rates or prostate symptom scores in patients with bladder outlet obstruction due to BPH (Liao and Kuo 2018; Nitti et al. 2013), though it improved urinary flow rates and total symptom scores following its add-on to α_1_-blockers in patients with mixed LUTS and α_1_-blocker-resistant symptoms (Kang and Chung 2020; Matsuo et al. 2016). Thus, careful assessment of target and off-target effects in preclinical studies and alignment with therapeutic concentrations is central to understand mechanisms of actions and to avoid false conclusions.

Consistent with our vibegron experiments, findings with mirabegron under the same conditions also indicated a lack of effects on voiding contractions at physiological concentrations (Huang et al. 2022c). The study addressed impacts of 1 µM and 10 µM mirabegron on carbachol-, methacholine- and EFS-induced contractions of human detrusor tissues (Huang et al. 2022c). Attenuations were lacking with 1 µM, suggesting that the maximum plasma levels of 137 nM are insufficient to inhibit voiding contractions in vivo (Huang et al. 2022c; Krauwinkel et al. 2012). Thus, both mirabegron and vibegron share similar patterns of action in vivo and in vitro, suggesting that improvements of storage symptoms without direct inhibition of cholinergic voiding contractions represent a class effect of β_3_-adrenergic agonists. Meanwhile, several mechanisms have been suggested to explain clinical effects of mirabegron in OAB, replacing the original assumption that inhibition of cholinergic detrusor contractions through activation of β_3_-adrenoceptors on bladder smooth muscle cells was responsible for symptom reduction (Igawa et al. 2019; Michel 2016). Improvements by mirabegron (and thus, by vibegron) may result from a suppression of the voiding reflex, caused by inhibition of microcontrations, or by activation of β_3_-adrenoceptors on mechanosensitive, afferent Aδ or C fibers in the bladder wall or on neurons of dorsal root ganglions (Aizawa et al. 2012, 2015; Sadananda et al. 2013). However, the maximum symptom improvement by mirabegron occurs after 4 weeks at the earliest, suggesting the involvement of long-term effects which may provisionally include changes in smooth muscle cell phenotype, or in expression of contractile proteins or receptors (Michel et al. 2025; Muderrisoglu et al. 2024). An involvement of the bladder trigone could be suspected as well, but β_3_-adrenoceptors or its ligands have never been explored in the human trigone (Hennenberg and Michel 2024). All these mechanisms are compatible with our current findings, including the inhibition of microcontractions, which seems possible with the relaxations of pretensions by 10–100 nM vibegron.

Increases in EC_50_ values for α_1_-adrenergic agonists in prostate tissues occurred with 10 µM vibegron across all three examined agonists and both tissue types, though not entirely consistently. Signs of antagonism or anticontractile effects were lacking with the lower concentrations, suggesting that binding to α_1_-adrenoceptors or improvements of voiding symptoms can not be expected in vivo with standard doses of vibegron. α_1_-Adrenoceptor antagonists are the fist-line option for medical treatment of voiding symptoms in BPH (Gravas et al. 2023), and are believed to improve symptoms by relaxation of prostate smooth muscle, or by blockade of neuronal α_1_-adrenoceptors. Using phenylephrine, methoxamine and noradrenaline, our findings suggest affinities of 1.78 µM, 1.86 µM and 9.77 µM in tissues from rPx, and 16 µM, 36 µM and 4.28 µM in laser-enucleated tissues. Whether affinities are in fact elevated in laser-enucleated compared to prostatectomized tissues, or whether this reflects zonal differences (rPx tissues were sampled from the periurethral zone, while tissues from HoLEP were morcellated parts from all ablated regions) remains open. About one third of patients treated for voiding symptoms with prescribed drugs are unresponsive to α_1_-blockers (Hennenberg and Michel 2024), so that different ligand affinities, as seen with vibegron may not be excluded. Consistent with effects on α_1_-adrenergic agoinsts, 10 µM vibegron but none of the lower concentrations reduced EFS-induced contractions of rPx tissues. While it may be concluded that this inhibition resulted from antagonism of α_1_, no such effect was observed on EFS-induced contractions in HoLEP tissues. This may again indicate differences in affinity to α_1_-adrenoceptors, or additional neuronal off-targets in rPx tissues that are missing in laser-enucleated tissues. On the other hand, it is noticeable that the EFS-induced contractions of laser-enucleated tissues were also very weak in the control groups, so that artifacts such as damage to the nerve remnants due to morcellation cannot be excluded.

Apart from technical issues, the two types of prostate tissue in our study and the patient cohorts are characterized by obvious differences. Our laser-enucleated prostate tissues represent advanced BPH, as the surgery for BPH is performed in patients showing mostly severe and medication-refractory voiding symptoms, or complications of BPH, or who are at high risk for complications (Keller et al. 2025a). In turn, prostate tissues from rPx without prior surgery for BPH represent milder stages of BPH, i.e. from patients without or with only mild to moderate voiding symptoms, and without complications of BPH and no need for BPH surgery. Based on the epidemiology of BPH and its age-dependent prevalence (Lepor 2004), BPH and voiding symptoms are common in patients undergoing radical prostatectomy for prostate cancer. Histologic BPH may occur in an estimated 80% of prostatectomized prostate cancer patients (Alcaraz et al. 2009; Orsted and Bojesen 2013). BPH is highly heterogenous, including stromal, glandular-epithelial and mixed hyperplasia, and each phenotype may cause voiding symptoms (Strand et al. 2017). Different tissue composition in rPx and laser-enucleated tissues, by divergent prevalence of stromal and glandular hyperplasia may account for different responses in organ bath experiments (Keller et al. 2025a; Keller et al. 2025b), including different sensitivity to vibegron. However, whether tissue composition in patients with surgical BPH is really different from other cohorts is not yet understood.

The main or single subtype mediating human prostate smooth muscle contraction is α_1A_. Competition binding assays suggested lacking binding of vibegron to α_1D_, but did not include α_1A_ (Brucker et al. 2022). Thus, effects seen with 10 µM on contractions by α_1_-adrenergic agonists were probably caused by antagonism of α_1A_. Contributions from β_3_-activation on prostate smooth muscle cells with subsquent contraction inhibition by cAMP formation seems unlikely, first as endothelin-1- and U46619-induced contractions remained unchanged by 10 µM vibegron, and secondly because anticontractile effects on α_1_-adrenergic prostate smooth muscle contractions have been observed with some, but not each examined β_3_-agonist (Huang et al. 2022b). Complementarily, stimulation with mirabegron also did not result in detectable cAMP formation in a cell culture model of human prostate stromal cells (Huang et al. 2021). Similar to our findings with vibegron, off-target antagonism of α_1_-adrenoceptors has been suggested for mirabegron, again with prostate tissues from radical prostatectomy (Huang et al. 2021). Signs of antagonism were largely absent at 1 µM mirabegron, but evident at 5 µM and pronounced at 10 µM (Huang et al. 2021). With K_i_ values of 0.437 µM for α_1A_-, 1.8 µM for α_1D_-, and 26 µM for α_1B_-adrenoceptors (Alexandre et al. 2016), the affinity of mirabegron for α_1_-adrenoceptors determined in biochemical competition assays appears provisionally higher as the affinity of vibegron estimated in our study. Antagonism of α_1_-adrenoceptors has been identified as a common off-target effect of β-adrenergic ligands, potentially mostly occuring with β_3_-agonists (Michel 2020). Contrary to initial assumptions, binding to α_1_ does not directly depend on phenylethanolamine moieties, since β_3_-agonists without such structures also exhibited such α_1_-adrenergic antagonism, even with comparatively high affinity, whereas not all β_3_-agonists with phenylethanolamine backbones exhibited such antagonism (Huang et al. 2022b). In silico investigations simulating the binding of mirabegron to a cryo-electron microscopy structure of α_1A_ revealed that the phenylethanolamine part indeed bound to transmembrane regions close to the binding pocket (though to positions not directly belonging to it), but that interactions of distal molecule parts with exosites at the receptor surface are obviously crucial as well (Huang et al. 2024). Specifically, exosite binding predominantly involved the distal anilino part of mirabegron, rather than its aminothiazol end (Huang et al. 2024). Similar interactions appear possible with vibegron, which contains a distal anilino part and a phenylethanolamine moiety as well, but remain subject to further investigation.

Our study design aimed to explore effects of vibegron in different human tissue types, and was therefore based on comparison of contractions in the presence of vibegron to contractions in vehicle controls without vibegron. Provisionally, our findings may point to slight differences in vibegron responses between tissues with advanced (laser-enucleated) and mild BPH (rPx). An influence of tumor infiltration can not be excluded in tissues from rPx and rCx, as these surgeries were performed for prostate or bladder cancer. Further conditions, such as inflammation and others may influence responses in the organ bath as well, but were not specifically analyzed in our study, where tissues were anonymized immediately after sampling. Unlike the two cohorts subjected to collection of prostate tissues, where population-specific characteristics on voiding symptoms and BPH are at least partly known, the presence and stages of OAB are unclear in our rCx cohort. A targeted assessment of vibegron responses in tissues from OAB in comparison to healthy patients may be of particular interest, and could include further study designs, such as comparison of contractions with vibegron between different patients groups, instead of comparisons between vibegron groups with vehicle controls. However, tissues from healthy patients, or bladder tissues from OAB patients without cancer are nearly unaccessible. Together, a generalization of our findings to healthy conditions, i.e. to persons without LUTS, without OAB or BPH, and without urogential cancers is not possible. Nevertheless, our data contribute to the understanding of mechanisms of β_3_-agonists in storage symptoms. Understanding of drug actions in general, and identification of their targets and mechanisms in preclinical studies is in general essential to foster advances in medicine and for clinical developments. Specifically, it emerges from post-approval research on β_3_-agonists, that microcontractions and neuronal mechanisms in the voiding reflex are suitable targets for OAB treatment (Michel et al. 2023), whereas direct inhibition of voiding contractions by anticholinergics was the prefered strategy for decades. β_3_-Agonists could replace anticholinergics as the first-line option in OAB treatment, as they are much better tolerated (Michel et al. 2023). Meanwhile, prescriptions for β_3_-agonists may already exceed those for anticholinergics, in at least some countries (Juliebø-Jones et al. 2025).

## Conclusions

Symptom improvements by vibegron in OAB may occur independently from inhibitions of voiding contractions in the detrusor. At concentrations in ranges of plasma levels, vibegron does not inhibit full cholinergic or neurogenic contractions in isolated human detrusor tissues from radical cystectomy for bladder cancer, but causes relaxations of mechanical pretensions. Prostate smooth muscle contractions remain unaffected as well at physiological concentration in tissues from radical prostatectomy for prostate cancer or from laser enucleation for BPH, suggesting that effects on voiding symptoms are unlikely. Off-target effects may include antagonism of muscarinic M_3_ receptors in bladder smooth muscle and of α_1A_-adrenoceptors in prostate smooth muscle, both with low affinity and not occuring with plasma concentrations.

## Data Availability

All data that support the findings of this study are included in this published article. Raw data are available from the corresponding author upon reasonable request.
